# Ionoregulatory and hematological parameters of *Triportheus albus* populations living in natural white- and blackwaters of the Amazon

**DOI:** 10.1007/s00360-025-01651-y

**Published:** 2026-01-29

**Authors:** Rafael Mendonça Duarte, Susana Braz-Mota, Maria de Nazaré Paula Silva, Guacira de Figueiredo Eufrasio Pauly, João Henrique Alliprandini Costa, Jhonathan da Mota Silva, Adalberto L. Val

**Affiliations:** 1 Laboratory of Ecophysiology and Molecular Evolution, Brazilian National Institute for Research of the Amazon (INPA), Manaus, Brazil; 2 Biosciences Institute, São Paulo State University - UNESP, Coastal Campus, São Vicente, SP Brazil

**Keywords:** Acid-base regulation, Hematological parameters, Na^+^/K^+^-ATPase, H^+^-ATPase, Physiological plasticity

## Abstract

In the Amazon basin, the white- and blackwater rivers have marked variability in their natural physicochemical characteristics, which impose differential physiological constraints that shape aquatic biodiversity. *Thriportheus albus* is a Characiform fish widely distributed throughout the different types of water in Amazon basin, with two distinct and isolated populations, a whitewater and a blackwater/clearwater population. In this study we compare blood biochemical, hematological parameters and the activity of the main ionoregulatory enzymes in gills and kidneys of *T. albus* populations from the blackwaters of Rio Negro and in the whitewater’s of Rio Solimões. *T. albus* from the blackwaters showed a higher hemoglobin concentration, hematocrit and levels of Na and Ca in blood compared to fish from the whitewater. Remarkably, the activity of both Na^+^/K^+^-ATPase and NEM sensitive-ATPase were significantly higher in the gills and kidneys of *T. albus* from blackwater. A Principal Component Analysis (PCA) revealed that up regulation of both transporters were closed related to increased blood parameters in *T. albus* from blackwater, suggesting that physiological adjustments in mechanisms for ionic uptake in gills and reabsorption in kidney aid fish to avoid internal disturbances in blood ionic levels, and optimize oxygen transport, to cope with the extremely low ionic content and high acidity found in Rio Negro. Altogether, these findings indicate that physiological adjustments in hematological parameters, and in the control of the gills and kidneys functions exert a central role in the adaptation of *T. albus* to the acidic ion-poor conditions of Amazonian blackwaters.

**Supplementary Information:**

The online version contains supplementary material available at 10.1007/s00360-025-01651-y.

## Introduction

The aquatic environment of the Amazon drains a total area of almost 7,000,000 km^2^ and has different geological and geomorphological properties, which explain the marked variability in the natural physicochemical and limnological characteristics in its water bodies (Junk et al. 2011). Sioli (1956) was the first to use optical (color and transparency) and chemical (pH and electrical conductance) properties to classify Amazonian rivers into three different types of water, the so-called whitewaters, blackwaters and clearwaters. Further studies revealed that these types of water display different physicochemical properties, especially in terms of alkali and alkali-earth metals, cationic and anionic content, total nitrogen and phosphorus, suspended sediment load and concentration of dissolved organic matter (Furch 1984; Furch and Junk 1997; Ríos-Villamizar et al. 2020), thereby generating pronounced environmental and ecological gradients that sustain the remarkable biodiversity of Amazonian aquatic system (Junk et al. 1983; Val and Almeida-Val 1995).

According to the general classification, the whitewater rivers have their origin in the Andes and are characterized by high amount of suspended solids, turbidity, nutrients (as N and P) and ionic content (in relation to the other Amazonian water types), conductivity (between 40 and 140 µS cm^− 1^), and a neutral pH. The blackwater rivers are recognized by their dark-colored waters with very low ionic content and primary productivity, and a high transparency (about 60–120 cm), since their drainage area is on the Precambrian Guiana shield, which is characterized by large areas of white sands (podzols). Perhaps the most remarkable property of blackwater rivers is the high amount of dissolved organic matter (DOM) (e.g., between 5 and 30 mgC/L), formally quantified as dissolved organic carbon (DOC), which is a complex matrix of compounds rich in humic and fulvic acids, and which are formed by the microbial processing of lignin-rich plant materials and the decay of organic remains of animals, giving these waters their characteristically high acidity (pH ranging from 3.5 to 5.0). The clearwater rivers have translucent waters (transparency greater than 150 cm) with a very low suspend solids content, ionic content and DOC, and a pH close to neutral (6.0–7.0), due to them draining the archaic Central Brazilian shield (Sioli 1956; Furch 1984; Furch and Junk 1997; Junk et al. 2011; Ríos-Villamizar et al. 2020). However, many rivers and streams in the Amazon basin can be classified as “mixed waters” owing to the influence of seasonal and spatial differences in the run-off of tributaries and by the oscillation of river levels (Junk et al. 2011; Souto et al. 2015; Ríos-Villamizar et al. 2020), thus promoting substantial limnological and ecological gradients that impose physiological constraints upon their aquatic communities (Junk et al. 1983; Petry et al. 2003; Cooke et al. 2012).

This seems particularly true for fish species with wide distribution throughout the aquatic ecotones of the Amazon, since they inhabit rivers with different types of water and make short transitions among them for foraging or for seasonal reproductive migrations (e.g., during the rising waters of the flood pulse) (Araújo-Lima & Ruffino 2003). For example, the Characiform fish *Triportheus albus* (Cope, 1872), popularly known as the freshwater sardine, is a benthopelagic species with an omnivorous feeding habit and a high ecological and socio-economic relevance, as it disperses seeds in flooded forests (Weiss et al. 2016) and is widely used as a source of protein by riverine populations – particularly in communities located in the whitewaters of the Solimões-Amazon system (Batista et al. 2012; Isaac et al. 2016). Genetic studies have assessed the populational structure of *T. albus* in the Central Amazon and support the distinction of two historically isolated populations, a whitewater and a blackwater clearwater population, with a very low level of admixture between whitewater and blackwater populations, thus indicating punctual migration events downstream from tributaries of the Negro River to the main channel of the Amazon River (Cooke et al. 2012; Hay et al. 2022). It is likely that the divergence between the two identified clades of *T. albus* (white and blackwaters ecotypes) is recent and are closely related to the final formation events of the Amazon River, occurred during the late Pliocene around 2.5 Ma (Cooke et al. 2012), where divergent selection between water colors led to the evolution of reproductive isolation (Schluter 2000). Indeed, *T. albus* (together with another congenerous species of the Triportheidae family) is a potamodromous fish that exhibits short- to long-distance migratory habits (100 km or less), transitioning between different types of water (Oliveira and Ferreira 2002; Araújo-Lima & Ruffino 2003; Lima& Araújo-Lima 2004), which requires a certain degree of physiological adjustments to maintain internal homeostasis under a significant gradient of environmental conditions.

In this context, some studies have assessed and annotated the function of candidate adaptive genes that are up-regulated in *T. albus* populations of different types of water, with special reference to gene-coding membrane components, acid-sensitive ion transport pathways, cytoskeletal/cell adhesion change, synthesis of proteins and light-sensitive photoreceptor pathways (Araújo et al. 2017; Hay et al. 2022). Specifically, a blackwater population of *T. albus* showed up-regulation of genes related to the control of ionic paracellular permeability in gills (such as gene coding for gap junctions, claudin, actin – actn4, and cell adhesion – itgb3b, and junction – DSP), for Ca^2+^ uptake (Ca^2+^-ATPase) and for the excretion of acid (H^+^/NH_4_^+^) and base (HCO_3_^−^) products of metabolism coupled to mechanisms for Na^+^ and Cl^−^ uptake, respectively (such as for Na^+^/K^+^-ATPase, NHE – slc9a6a, v-type H^+^-ATPase – ATP6V0A2, rhesus protein – rhcg1, bicarbonate transporters – slc4a4b and slc26a4) (Araújo et al. 2017). Interestingly, this evidence supports previous physiological studies that suggested that the control of gill functions, particularly the diffusive ionic permeability and transcellular uptake of ions, is the key physiological adaptation for fish to thrive in the harsh environment of blackwaters (Gonzalez et al. 2002, 2006; Wood et al. 2003; Morris et al. 2021). Therefore, as pointed by Araújo and collaborators (2017), *T. albus* populations might display phenotypic plasticity in mechanisms related to the maintenance of internal acid-base and ionic homeostasis, which seems to be directly controlled by physicochemical gradients in different types of water, where fish are required to display differential and unique adaptations to cope with distinct environmental pressures.

With this background in mind, in this study, we tested the hypothesis that *T. albus* exhibits differential responses in branchial and renal mechanisms related to ionic uptake and acid-base regulation among populations from blackwaters and whitewaters, avoiding internal disturbances in ionic homeostasis and in blood respiratory parameters. Specifically, the objectives of this study were (1) to compare biochemistry and hematology parameters, as well as to determine the activity of the main ionoregulatory enzymes in gills and kidneys of two distinct populations of *T. albus* in the Central Amazon that live in the blackwaters of Rio Negro and in the whitewaters of Rio Solimões; and (2) to identify the key physiological and biochemical adjustments in *T. albus* populations that enable their physiological plasticity to facilitate adaptation to aquatic environments with significantly distinct physicochemical properties. To address this issue, we measured and compared biochemistry (glucose, Na, K and Ca) and hematology parameters (Hct and Hb), as well as Na^+^/K^+^-ATPase, NEM-sensitive ATPase and carbonic anhydrase activities in gills in kidneys between blackwater and whitewater populations of *T. albus*.

## Materials and methods

### Ethics statement

The permit for the collection of the biological material to carry out the research was authorized by the Brazilian Institute of Environment and Renewable Natural Resources (IBAMA/SISBio) under number 29837-24. The collection, care and use of experimental animals were in accordance with the Brazilian Ethics Committee on Animal Care (CONCEA) guidelines (RN 61/2023) and was previously approved by the Ethics Committee on the Use of Animals (CEUA) at INPA (protocol number: 004/2018).

### Study area and fish collection

The Amazonian sardines, *Triportheus albus*, weighing 33.69 ± 2.74 g and with a total length of 16.21 ± 0.43 cm (mean ± SEM), were collected in the blackwaters of the Rio Negro, within the Anavilhanas National Park (S 02°43’10.0’’; W 060°45’20.1’’), next to the floating base of the Chico Mendes Institute for Biodiversity Conservation (ICMBio) in the Lago do Prato (Novo Airão, Amazonas) – and in the whitewaters of the Rio Solimões (S 03°21’08.4’’; W 060°11’25.7’’), close to Maniquiri, Amazonas. This study was conducted during a 15-day boat expedition, between November and December 2023, which was funded by the ADAPTA-II project. This period represents the first month of rising water levels after the greatest drought in history of the Rio Negro, which was at its lowest in October 2023 (Espinoza et al. 2024). At both sites, twelve individuals of *T. albus* (*N* = 12) were captured during the morning with net traps (6 sides, diagonal: 35 × 35 / 80 × 80 cm approx.) and then kept in a 400-L plastic pool with the same water as the sampling site and with vigorous aeration. During this acclimation period water temperature in holding tank was similar to those seen in natural water from rivers (i.e. ranging from 30.3 to 35.3 °C in blackwater and 30.3–33.2 °C in whitewater). The fish were kept under these conditions for 24 h prior to the analysis so as to reduce the stress of capture. During this period, the water was partially renewed four times (around 25% each time) to avoid the accumulation of feces and nitrogen waste in the pool. The fish were not fed and no mortality was recorded over the 24 h in the plastic pool. For 5 days at each sample site, water quality parameters, such as oxygen concentration (mg/L), pH and conductivity (µS/cm), were monitored twice a day using a multi-parameter (YSI pro plus), while the temperature of the water was recorded using a datalogger (Pendant MX Temp HOBO) (± 0.5 °C), at an interval of every 10 min, just below the surface (~ 5 cm) and at the bottom (around 2.8 m).

### Blood collection and biochemistry and hematological parameters

Twenty-four hours after field collection, the fish were removed from the plastic pool and anesthetized in a neutral buffered solution of tricaine methanesulfonate (1 g/L of MS-222 and 2 g/L of NaHCO_3_, Sigma-Aldrich) for taking blood samples. Blood was drawn from the caudal vein (around 0.5 mL per fish) using a heparinized 3.0 mL syringe attached to a 22-gauge needle. To measure the hematocrit (Hct), hemoglobin concentration ([Hb]), concentration of K, Na, Ca and glucose in blood of *T. albus*, an iSTAT portable clinical analyzer (Abbott Point of Care Inc., Princeton, NJ, USA) was used. The i-STAT system measures blood parameters directly in whole blood, not plasma, because its method relies on the electrical conductivity of blood. While differences may arise due to the presence of blood cells, the use of iStat for whole blood analysis is well established and provides reliable results, especially under field work conditions, with a strong correlation to plasma values reported in the literature, with a strong correlation to plasma values reported in the literature (Stoot et al. 2014). Immediately after the procedure, the blood samples were stored on ice and an aliquot (~ 0.095 mL) was loaded into an iSTAT CG8 + cartridge within 5 min of sample collection.

### Enzymatic assays

After blood collection, the fish were euthanized and the gill and kidney samples were obtained, rinsed in saline solution (0.9% NaCl) and gently blotted on filter paper to minimize excess blood and immediately frozen and maintained in liquid nitrogen until returning from the boat expedition. The samples were then stored at -80 °C in a freezer in the laboratory until the analysis for the determination of the activity of Na^+^/K^+^-ATPase (NKA), NEM-sensitive ATPase (N-ethylmaleimide (NEM)-sensitive proton-translocating adenosine triphosphatase) and carbonic anhydrase (CA).

In summary, the activities of both NKA and NEM-sensitive ATPase in the gills and kidneys of the specimens of *T. albus* were determined using the protocol described by Kültz and Somero (1995), which is based on the oxidation of NADH in an enzymic reaction coupled to the hydrolysis of ATP. The assay is based on the inhibition of NKA activity by ouabain (2 mM), and NEM-sensitive ATPase by N-ethylmaleimide (NEM, 2 mM). Aliquots of gill and kidney samples were homogenized (1:10 w/v) in buffer (pH 7.5) containing (in mM): sucrose 150, imidazole 50, EDTA 10 and deoxycholic acid 2.5, and centrifuged at 2,000 *g* for 7 min at 4 °C. Supernatants were added to a reaction mixture containing (in mM): imidazole 30, NaCl 45, KCl 15, MgCl_2_ 3.0, KCN 0.4, ATP 1.0, NADH 0.2, fructose-1,6-bisphosphate 0.1, PEP 2.0, with 3 U mL^− 1^ pyruvate kinase and 2 U mL^− 1^ lactate dehydrogenase. Four replicates of each sample were run with and without the inhibitors for NKA and NEM-sensitive ATPase (i.e., ouabain and NEM, respectively). Decaying of absorbance was determined spectrophotometrically over 10 min at 340 nm (SpectraMax M2, Molecular Devices Inc., Sunnyvale, CA, USA). The final activity of both NKA and NEM-sensitive ATPase activities were calculated via the differences between total activity and activities with ouabain and NEM inhibitors, respectively, and expressed as mmolATP/h/mg of protein. Total protein in the gill and kidney samples were determined spectrophotometrically at 595 nm according to the colorimetric assay described by Bradford (1976) using a bovine serum albumin as standard.

The activity of CA in the gills and kidneys of the *T. albus* specimens was quantified according to the assay described by Vitale et al. (1999), based on Henry (1991). Thus, the samples were homogenized (1:10 w/v) in phosphate buffer (10 mM, pH 7.4) and centrifuged at 2,000 *g* for 5 min at 4 °C. The supernatants (50 µL) were added to 7.5 mL of reaction buffer (pH 7.4) containing (in mM): mannitol 225, sucrose 75, and trisphosphate 10 and 1 mL of cold distilled water saturated with CO_2_. Immediately after the addition of the CO_2_ -saturated water, the reduction in pH was followed for 20 s, with pH readings every 4 s. The pH was measured using a benchtop pH meter (OHAUS ST2200-F). All assays were carried out at a controlled temperature of 2.5 °C. Changes in pH were converted to hydrogen ion concentrations [H⁺] using the equation [H⁺] = 10^(-pH). Carbonic anhydrase specific activity (CAA) was calculated as: CAA = [(CR/NCR) − 1]/mg of total protein in the sample (CR: catalyzed rate and NCR: non-catalyzed rate). Total protein in the gill and kidney samples were determined spectrophotometrically at 595 nm according to the colorimetric assay described by Bradford (1976) using a bovine serum albumin as standard.

### Statistical analysis

All the data were assessed for normality and homogeneity of variance prior to the statistical analyses and were presented as mean ± standard error of the mean. Student’s t tests were conducted to identify differences in water chemistry between the blackwaters and whitewaters of the Rio Negro and Rio Solimões, respectively, as well as to verify differences in biochemistry and hematology parameters, and ionoregulatory enzymes of the *T. albus* specimens collected in both water types. The level of significance (α) in all the tests was 0.05. In addition, we performed a principal component analysis (PCA) to investigate the similarity and distribution of the physiological and biochemical variables of the *T. albus* populations from the blackwater and whitewater. The analysis was conducted using R (version 4.2.1), and we utilized the *factoextra* package (Kassambra & Mundt 2020). Before PCA analysis, all environmental variables were standardized (mean = 0, standard deviation = 1) to ensure comparability among variables measured on different scales. The data was standardized to ensure comparability between variables with different units. For the PCA, two factors were extracted considering eigenvalues higher than 1.0 (Kaiser’s criteria) and a component-loading cut-off of 0.45 was used to select the variables that are considered for inclusion into the two factors (Tabachnick and Fidell 2013).

## Results

The main physicochemical parameters of the blackwater and whitewater rivers characterized in this study are shown in Table 1. As previously mentioned, the whitewater from the Solimões River presented a significantly lower oxygen concentration, higher pH (+ 1.35 units of pH) and conductivity (8.8 times) in comparison to the acidic, ion poor blackwaters of the Rio Negro. Regarding water temperature, there was no significant difference in average temperature comparing surface and bottom temperatures, within and among both types of water, but the temperature at the surface of the Negro River can be 2 °C higher than in the Rio Solimões during the warmest periods of the day (Supplementary material – S1).

**Table 1:** Measured water chemistry in natural black and whitewaters from Negro river (Novo Airão) and Solimões river (Manaquiri), respectively

	O_2_ (mg/L)	pH	Condutivity (µS/cm)	Temperature (^o^C)		Average temperature (^o^C)
				Surface	Bottom	
Blackwater	6.53 ± 0.32	4.67 ± 0.13	12.14 ± 0.73	31.99 (35.35–30.33)	31.11 (31.92–30.20)	31.55 ± 0.02
Whitewater	5.34 ± 0.31*	6.02 ± 0.31*	106.29 ± 12.01*	31.77 (33.16–30.28)	30.92 (31.49–30.33)	31.05 ± 0.01

Asterisks represent a statistically significant difference in water parameter between both types of water (*n* = 12; Two-tailed P-value < 0.018)

Temperature (^o^C) from surface and bottom are represented as mean (maximum and mininum) measured over five days at each sampling point

**Fig. 1:**
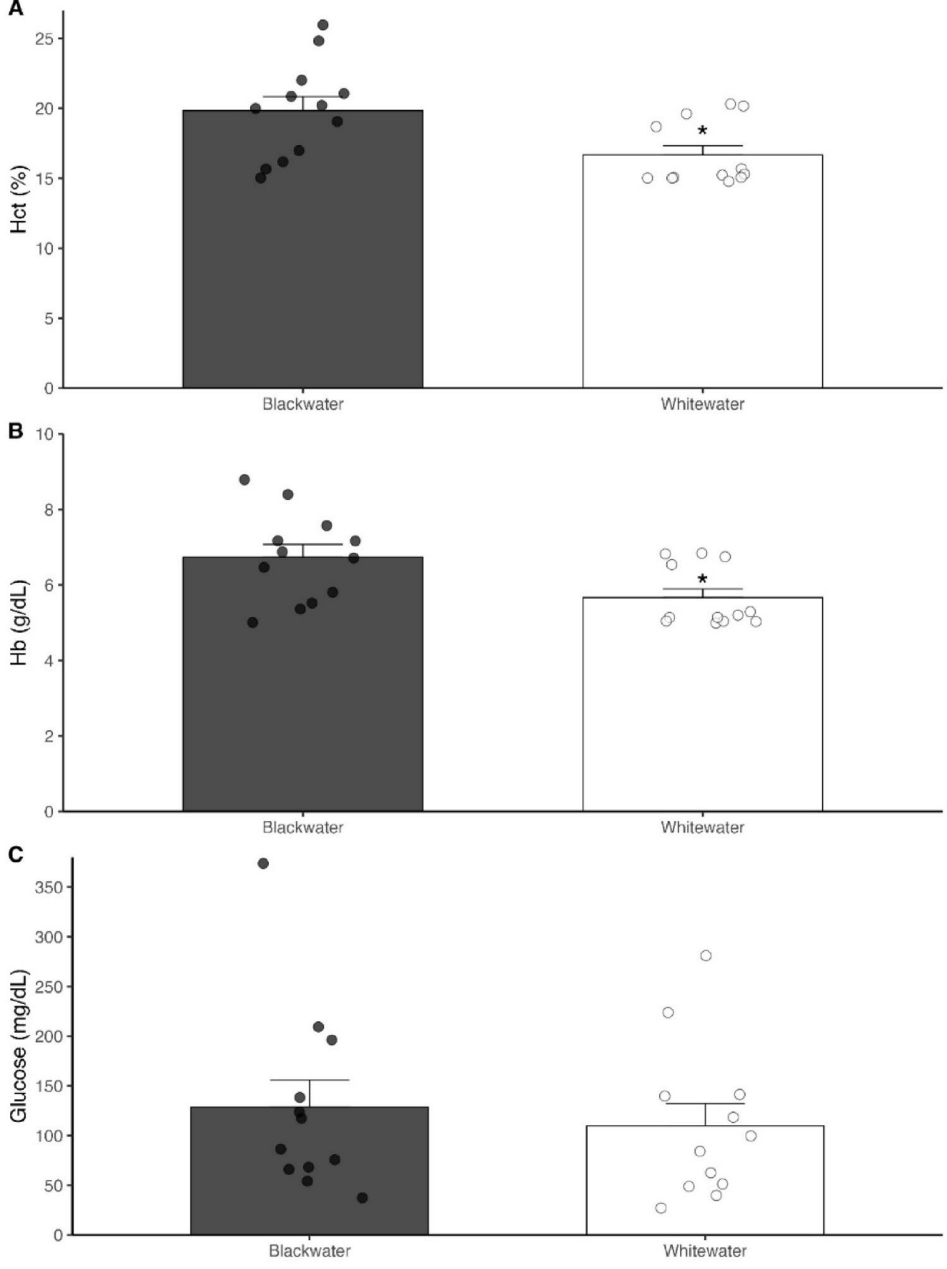
Hematocrit (%) (**A**), and concentration of hemoglobin (g/dL) (**B**) and glucose (mg/dL) (**C**) in blood of *Triportheus albus* collected in in Rio Negro blackwater (Anavilhanas/AM) and and in Rio Solimões whitewater (Manaquiri/AM). The dots represent the value of each individual collected in both types of water (*N* = 12 per water type. Asterisk represents significant statical differences in between both types of water (*N* = 12; Two-tailed P-value < 0.05)

**Fig. 2:**
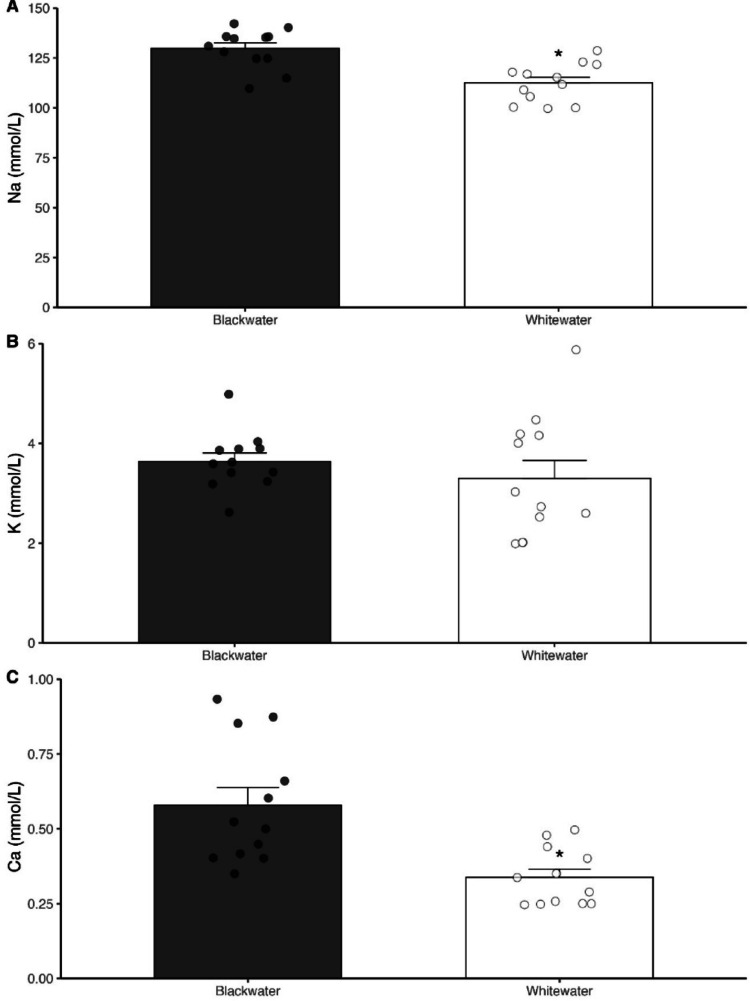
Concentration of sodium (**A**) potassium (**B**) and calcium (mmol/L) (**C**) in blood of *Triportheus albus* collected in in Rio Negro blackwater (Anavilhanas/AM) and and in Rio Solimões whitewater (Manaquiri/AM). The dots represent the value of each individual collected in both types of water (*N* = 12 per water type. Asterisk represents significant statical differences in between both types of water (*N* = 12; Two-tailed P-value < 0.05)

The values of biochemistry and hematological parameters in the blood of the *T. albus* specimens measured by the clinical analyzer are shown in Figs. 1 and 2. The type of water does not affect the concentration of glucose (*P* = 0.603) (Fig. 1C) and K (*P* = 0.402) (Fig. 2B) in the blood of fish. However, the *T. albus* specimens collected in the blackwater presented significantly higher levels of hematocrit and hemoglobin concentration (*P* = 0.016) (Fig. 1A and B), as well as of Na^+^ and Ca^2+^ (*P* = 0.001) (Fig. 2A and C) in their blood.

In the gills of the fish from the blackwaters, the activity of both Na^+^/K^+^-ATPase and NEM-sensitive ATPase was significantly higher (*P* = 0.02) (1.5 and 3.3 times, respectively) compared to fish from the whitewaters (Fig. 1A and B), while no differences in the activity of CA (*P* = 0.386) were seen in the gills of the fish from both types of water (Fig. 1C). Similarly, the activity of both NKA and NEM-sensitive ATPase were substantially higher (3.0 and 5.5-fold) in the kidneys of the *T. albus* specimens from the blackwater (*P* < 0.001) than those from the whitewater (Fig. 2A and B). There is no difference in CA activity (*P* = 0.488) in the kidneys of the fish from either type of water (Fig. 2C).

For an integrated analysis, the entire dataset was used to perform a PCA-factor analysis, which is shown in Supplementary material (S2). The first two principal factors extracted by the PCA explained over 55% of data variance, and biological variables with loadings higher than 0.45 were considered significant in the extracted factors of the PCA. Thus, the first factor (PC1) explained 35.7% of data variation and showed a positive association between Hct, [Hb], Na and Ca levels, and the activity of NKA and NEM-sensitive ATPase in both gills and kidneys. Thus, the PCA-factor analysis divided the data set into 2 well defined clusters by type of water indicating that these parameters (Hct, [Hb], Na, Ca and NKA and NEM-sensitive ATPase in both gills and kidneys) were higher in the fish collected from the blackwaters, with significant lower values seen in the fish from the whitewaters (Supplementary material – S2). On the other hand, the second factor (PC2) explained almost 20% and demonstrated a positive correlation of CA in the gills and Hct, [Hb] and glucose, which were negatively correlated to Ca levels in blood. However, in the second factor, there is no clear evidence for grouping fish from blackwaters with fish from whitewaters, indicating that the correlation among these physiological responses of *T. albus* might not directly be associated with differences in composition between both water types.

## Discussion

The results of our study revealed that the Amazonian water types modulated hematological and ionic parameters in the blood of *T. albus*, as seen as by higher hematocrit and hemoglobin concentrations, as well as in Na and Ca levels, in fish from the blackwater population (Figs. 1 and 2). The rise in both hematocrit and hemoglobin concentration (and sometimes in the number of red blood cells – RBC) is a well-documented hematological response to acute exposure to acid ion poor waters that is seen in many freshwater fish, e.g., *Oncorhynchus mykiss* (Milligan and Wood 1982), *Prochilodus scrofa* (Carvalho & Menezes 2006) and *C. macropomum* (Aride et al. 2007). These hematological responses are usually associated with a shift of fluid volume from extracellular to intracellular compartments, resulting in increased Hct, Hb and RBC, as a result of severe reductions in plasmatic ionic levels induced by the higher H^+^ content in water on ionoregulatory mechanisms in the gills of fish (Milligan and Wood 1982; Wood 2001). It is unlikely to be the case of *T. albus* living in blackwater since this population had Na and Ca levels of around 15% and 10% higher than those seen in fish from whitewater, respectively. Overall, these findings suggest a slight blood viscosity (hemoconcentration) in *T albus* living in blackwater, which can be explained by a greater control of the branchial permeability, reducing water gain by osmosis, and/or through a greater active of transcellular transport mechanisms (in gills and kidney) providing the electrochemical gradient necessary for the maintenance of Na/Ca uptake from a more acidic and diluted medium (see discussion below). Despite slightly higher oxygen availability, blackwaters present challenges such as low ion concentrations, high levels of dissolved organic carbon (DOC), and reduced buffering capacity, all of which can affect gas exchange and acid-base balance. Thus, the observed increases in Hct and Hb may also represent adaptive physiological adjustments for the optimization of O_2_ delivery to tissues, owing to the maintenance of efficient physiological homeostasis in a chemically complex and potentially stressful habitat.

In this study we observed higher levels of Na and Ca in the blood of the *T. albus* from the blackwaters (pH 4.7), compared to the fish from the whitewaters (pH 6.0). A similar result was seen for juvenile pirarucu (*Arapaima gigas*), whereby smaller fish (~ 200 g) displayed Na levels that were 30% higher following 7-day acclimation to acidic blackwater than for whitewater (Ramos et al. 2014). This findings contrast with the general models for ionic and osmoregulation in freshwater fishes at acidic conditions, where reduced levels of ions (e.g., Na^+^ and Cl^−^) in the blood and plasma are commonly reported, as a result of either the disruption in transcellular uptake mechanisms and/or loss of control of diffusive paracellular permeability in the gills, which seems to occur in a species-specific manner (Gonzalez et al. 2006; Kwong et al. 2014; Morris et al. 2021). In these regards, it is widely recognized that freshwater teleost fishes have a tighter gill epithelium, which is generally linked to the properties of the tight junction (TJ) proteins between gill epithelial cells, that limit passive ion loss and contributes to the overall maintenance of ionic and osmotic homeostasis of fishes in a hypoosmotic environment (reviewed by Chasiotis et al. 2012). For example, increased transcripts abundance of different isoforms of occludin and claudin were seen in gills and kidney of goldfish (*Carassius auratus*) following long-term acclimation to ion poor water (Chasiotis et al. 2009; 2012), as well as in different tissues of zebrafish exposed to acidic water (Kumai et al. 2011). Interestingly, Araújo et al. (2017) demonstrated that several genes encoding for tight junction proteins (e.g. claudin proteins, actinin alpha 4 – actn4, integrin beta – itgb3b, desmoplakin – DSP, and gap junction proteins) are up-regulated in gills of *T. albus* living in blackwater, compared with animals from whitewater population, suggesting a strong environmental modulation of blackwater conditions on the control of intrinsic branchial permeability of *T. albus*.

These findings might be associated to the presence of high amounts of the darker DOC from the Rio Negro, which is rich in humic and fulvic-like compounds, and which have been recognized to confer exceptional properties to this DOC source, such as the ability to attenuate ionoregulatory disturbances induced by low pH (Wood et al. 2011; Duarte et al. 2016; Morris et al. 2021). Examination of the protective action of DOC from the Rio Negro against disturbances mediated by low pH on physiological gill functions revealed that diffusive paracellular losses of Na^+^ and Cl^−^ were markedly reduced in Amazonian stingrays (*Potamotrygon* sp) and in *Geophagus* sp., when the fish were exposed to water from the Rio Negro with a pH of 4.0 compared to reconstituted water without DOC (Gonzalez et al. 2002; Wood et al. 2003). The protective physiological effects of DOC are believed to occur via the action of DOC molecules stabilizing paracellular junctions at low pH, which makes the gills more tight to ionic diffusive losses. As a result, a greater Na^+^ to Cl^−^ permeability ratio in gill membranes is expected, which results in hyperpolarization (i.e., a more negative transepithelial potential – TEP), which also would help fish to maintain Na^+^ uptake in acidic waters (Wood et al. 2011; Morris et al. 2021). Thus, further evaluations of unidirectional ionic fluxes in the gills of the *T. albus* population of the blackwaters would help to understand the effect of DOC from the Rio Negro on the control of paracellular permeability at low pHs, and determine whether this physiological response is the cause of the higher levels of Na and Ca seen in the blood of *T. albus* in this study. In addition, it can help to reveal if DOC is, in fact, responsible for the up regulation of genes encoding for paracellular junctions (Araújo et al. 2017), which seems to be a key physiological mechanism of the adaptation of *T. albus* to the conditions of the blackwaters.

Perhaps the most surprising finding of the present study was the significantly higher activity of both NKA and NEM-sensitive ATPase in gills and kidney, but not in CA activity, of the *T. albus* population from the acidic blackwater. During exposure to acidic waters, the excess H^+^ in the surrounding environment can inhibit ionic uptake in fish gills, which, in association with the stimulation of diffusive losses, can decrease plasmatic ionic levels. However, there is consistent evidence that some acidophilic fish species can maintain, or even increase, Na^+^ uptake at low pHs (pH 3.25–4.0) in standard or reconstituted water, as seen for zebrafish (Kumai et al. 2011), tambaqui (Gonzalez et al. 2021) and different species of Amazonian tetras, such as *Paracheirodon axelrodi*, *P. innesi* and *Gymnocorymbus ternetzi* (Gonzalez et al. 2006). Higher activities of NKA and NEM-sensitive ATPase are expected in fishes inhabiting extremely diluted environments, since the reduction in external ion concentrations requires the up regulation of branchial ion transporters to sustain transcellular Na⁺ and Cl⁻ uptake. This mechanism is well recognized as a common compensatory response to ionic dilution and helps stabilize internal homeostasis under low ion dissolved conditions (Kumai et al. 2011; Kwong et al. 2014). For example, in zebrafish, the stimulation of Na^+^ uptake after 5- to 7-day exposure to pH 4.0 seems to be directly associated with the activation of H^+^-ATPase, based on the up-regulation of zatp6v0c gene expression in gills (Yan et al. 2007), and also by NHE3 transporter, once the treatment with NHE-selective inhibitor (5-(N-ethyl-N-isopropyl)amiloride) or NHE3b knockdown prevented the acidic-stimulation of Na^+^ (Kumai and Perry 2011), indicating an important role of both transporters in Na^+^ homeostasis of fish under acidic conditions.

In addition, the Rio Negro DOC seems to favor the maintenance of Na^+^ uptake in acidic waters, as previously reported for *Corydoras julii*, *Carnegiella strigata* (Gonzalez et al. 2002) and *Potamotrygon* sp (Wood et al. 2003) exposed to low pHs in natural Negro River water, as well to zebrafish, for which DOC increased Na^+^ uptake rates and helped the fish to keep it coupled to ammonia excretion during exposure to pH 4.0 (Duarte et al. 2016). Interestingly, the DOC concentration of the Rio Negro and Rio Solimões increased the activity of both NKA and NEM-sensitive ATPase in the gills of zebrafish acclimated to DOC in water with a neutral pH, which was maintained at high rates after 3 h of exposure to a low pH (Sadauskas-Henrique et al. 2021). These findings support the assumption that DOC could stimulate the activity of NKA and NEM-sensitive ATPase in the gills of *T. albus*, which would help fish to kept Na^+^ uptake and sustain higher ionic levels in blood and cope with the ionoregulatory challenges imposed by the acidic conditions of blackwater.

Although gills are recognized as the main organ responsible for both ionoregulation and acid-base regulation of freshwater fish (Evans et al. 2005), the increased renal activity of both NKA and NEM-sensitive ATPase (on average 3-fold) in *T. albus* population from the blackwaters suggest a central role of kidney in adaptation to acidic ion poor conditions of Rio Negro. In vertebrates, one of the main functions of the kidney is intimately related to the control of blood pH, which occurs through the regulation of excretion rates of acid (H^+^ or NH_4_^+^) and base (HCO_3_^−^ or OH^−^) equivalents (Randal et al. 1996; Perry & Gilmour 2006), but in freshwater teleost fishes kidney also play an accessory role in the reabsorption of major ions – approximately 95% of the NaCl entering the glomerular filtrate – contributing to the maintenance of the ionic homeostasis in fish under hypoosmotic conditions (Perry et al. 2003; Takvam et al. 2023). Few studies have addressed the renal functions in Amazonian fish, but high urine flow rate (UFR) associated with both high Na^+^ reabsorption and a high ammonia excretion rate have previously been reported (Randal et al. 1996; Wood et al. 2017; Wood et al. 2020). These high reabsorption rates seem to be dependent on the high activity of NKA and NEM-sensitive ATPase, which are essential for generating the necessary electrochemical gradients that power the whole process (Perry et al. 2003; Takvam et al. 2023). To Amazon fishes, higher levels of NKA were measured in the kidneys of pirarucu (Wood et al. 2020) – in relation to the gills (10-fold), which is similar to what was found in *T. albus* from whitewater; whilst a 20-times higher activity of NKA in the kidneys in relation to the gills was observed in fish from blackwater. It was also observed an higher NEM sensitive ATPase activity in the gills (3.3-fold) and kidneys (5.5-fold) in the *T. albus* specimens from the blackwaters when compared to those from the whitewaters, which were on average 4.7-fold higher in the kidneys in relation to the gills in the fish from both types of water, which exceeds the ratio previously seen to the pirarucu (2.6-fold) (Wood et al. 2020). In addition, a marked increase in both NKA activity and in occludin expression were seen in kidney of goldfish following the acclimation to ion poor water, indicating that higher ionic reabsorption associated with reduced permeability of segments of nephron is essential to maintaining salt and water balance in ion-poor surroundings (Chasiotis et al., 2009). Therefore, it is likely that the up regulation of both NKA and NEM-sensitive ATPase in kidney contribute for a higher renal ionic reabsorption that, in association with increased activity of both transporters in gills, help *T. albus* to sustain higher ionic (Na and Ca) levels in blood against more unfavorable gradients from blackwaters, in comparison with *T. albus* population living in whitewaters.

Ultimately, to gain a deeper understanding of the effects of black- and whitewater acclimations on physiological and biochemical responses among *T. albus* populations, we used a principal component analysis that certified two separate clusters by the type of water (Supplementary material – S2). The first axis extracted by PCA pointing to a close association between Hct, [Hb], Na and Ca levels, and the activity of NKA and NEM-sensitive ATPase in both gills and kidneys, which were higher in *T. albus* population from blackwater, indicating physiological adjustments in ionoregulatory functions of gills and kidney aid fish to avoid internal disturbances in blood ionic levels, and optimize oxygen transport, to cope with the extremely low ionic content and high acidity found in Rio Negro. Given that for the adaptation to environments with distinct environmental pressures, as seen among Amazon water types, adjustments in physiological traits are required for improving organismal function and/or overall fitness (Kelly et al. 2012), the physiological and biochemical responses displayed by *T. albus* seems to be key adaptations to thrive in the acidic, ion-poor and high DOC conditions of the Rio Negro blackwater.

## Conclusions

The results of our study reinforce the findings of Araújo et al. (2017) and it should be highlighted that *T. albus* displays differential physiological responses in order to live in the different water types of the Amazon. Physiological adjustments in hematological and ionic parameters of the blood, and in mechanisms associated with ionic uptake in the gills and reabsorption in the kidneys seems to play a fundamental role in the local adaptation of *T. albus* to the environmental conditions found in the blackwaters of the Rio Negro. Given that the physiological adjustments displayed by the blackwater population of *T. albus* involve a high metabolic cost, the potential trade-offs for the acclimation to blackwater conditions – especially in the current scenario of global warming and increased contamination in Amazonian waters (Val et al. 2022; Braz-Mota and Val 2024) – should be investigated in the future.

**Fig. 3:**
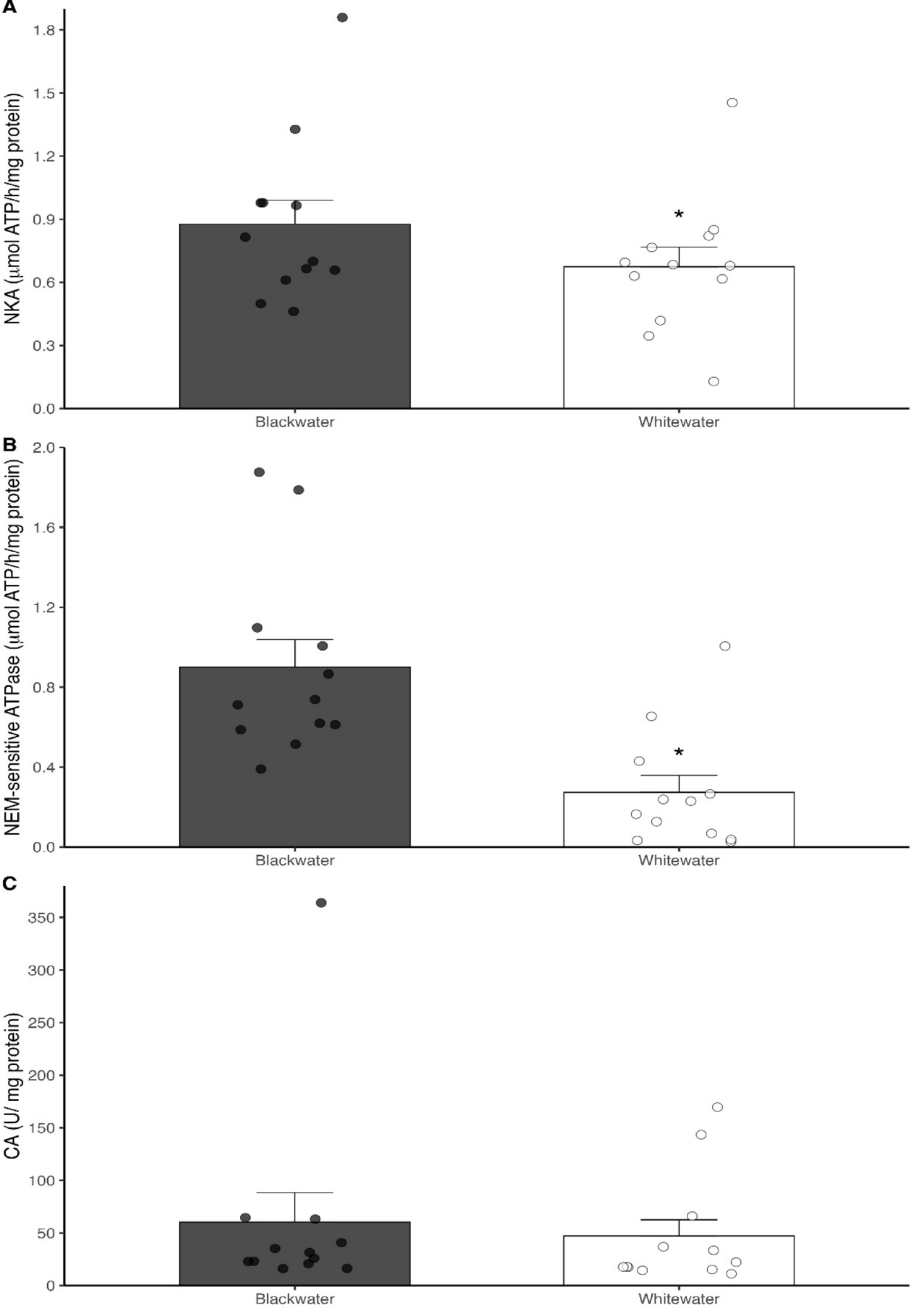
Activity of Na^+^/K^+^-ATPase (NKA) (**A**), NEM-sensitive ATPase (**B**) and carbonic anhydrase (CA) (**C**) in gills of *Triportheus albus* collected in in Rio Negro blackwater (Anavilhanas/AM) and and in Rio Solimões whitewater (Manaquiri/AM). The dots represent the value of each individual collected in both types of water (*N* = 12 per water type. Asterisk represents significant statical differences in between both types of water (*n* = 12; Two-tailed P-value < 0.05)

**Fig. 4:**
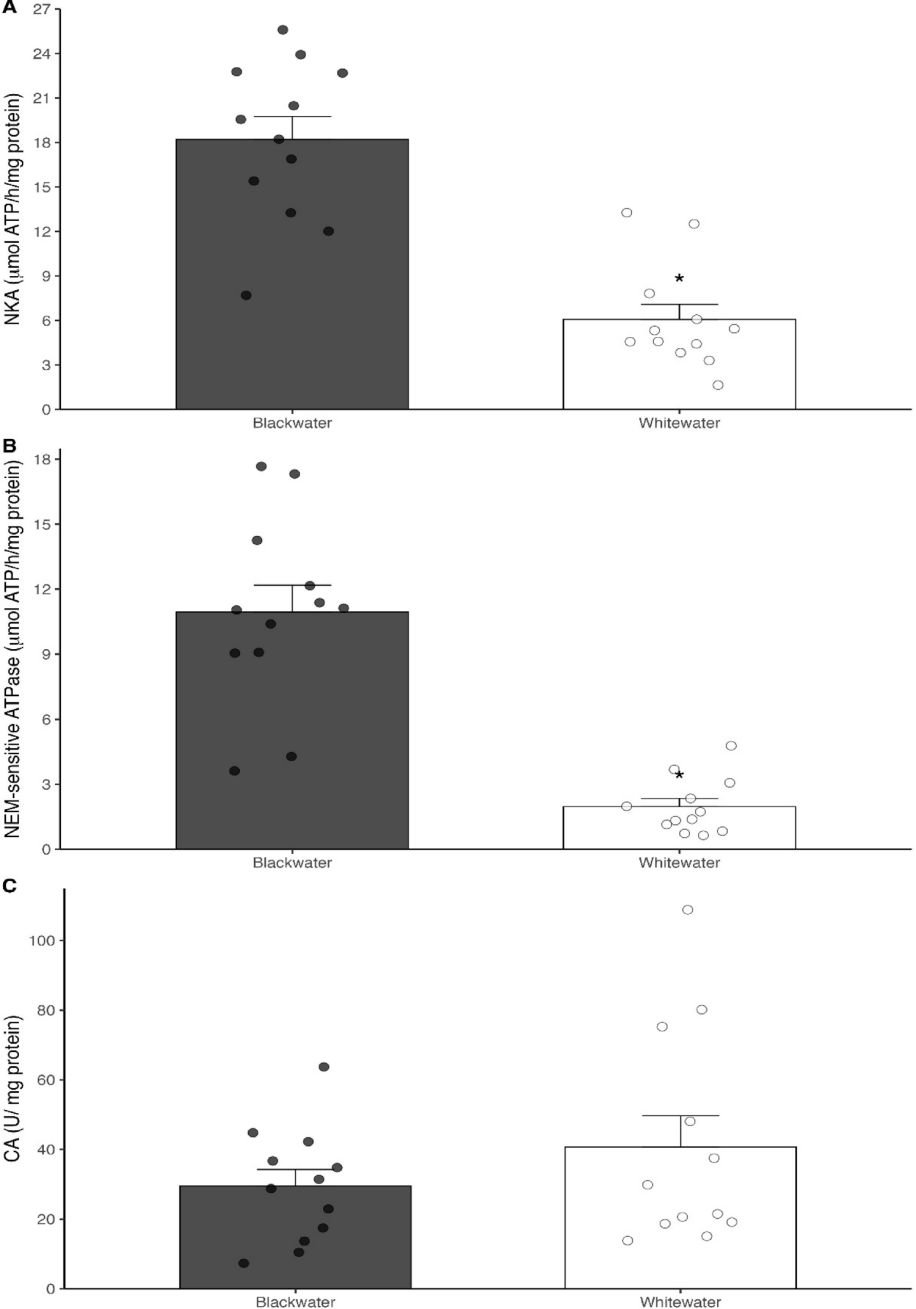
Activity of Na^+^/K^+^-ATPase (NKA) (**A**), NEM-sensitive ATPase (**B**) and carbonic anhydrase (CA) (**C**) in kidney of *Triportheus albus* collected in in Rio Negro blackwater (Anavilhanas/AM) and and in Rio Solimões whitewater (Manaquiri/AM). The dots represent the value of each individual collected in both types of water (*N* = 12 per water type. Asterisk represents significant statical differences in between both types of water (*n* = 12; Two-tailed P-value < 0.05)

## Supplementary Information

Below is the link to the electronic supplementary material.

Supplementary Material 1
