# Supplementary material for: Ionoregulatory and hematological parameters of Triportheus albus populations living in natural white- and blackwaters of the Amazon
Source: J Comp Physiol B. 2026 Jan 29;196(1):67–79. doi: 10.1007/s00360-025-01651-y (PMC12935810; doi:10.1007/s00360-025-01651-y)
Supplement: Supplementary file 1 — Supplementary Material 1 [file 360_2025_1651_MOESM1_ESM.docx]

Supplementary material

**S1**


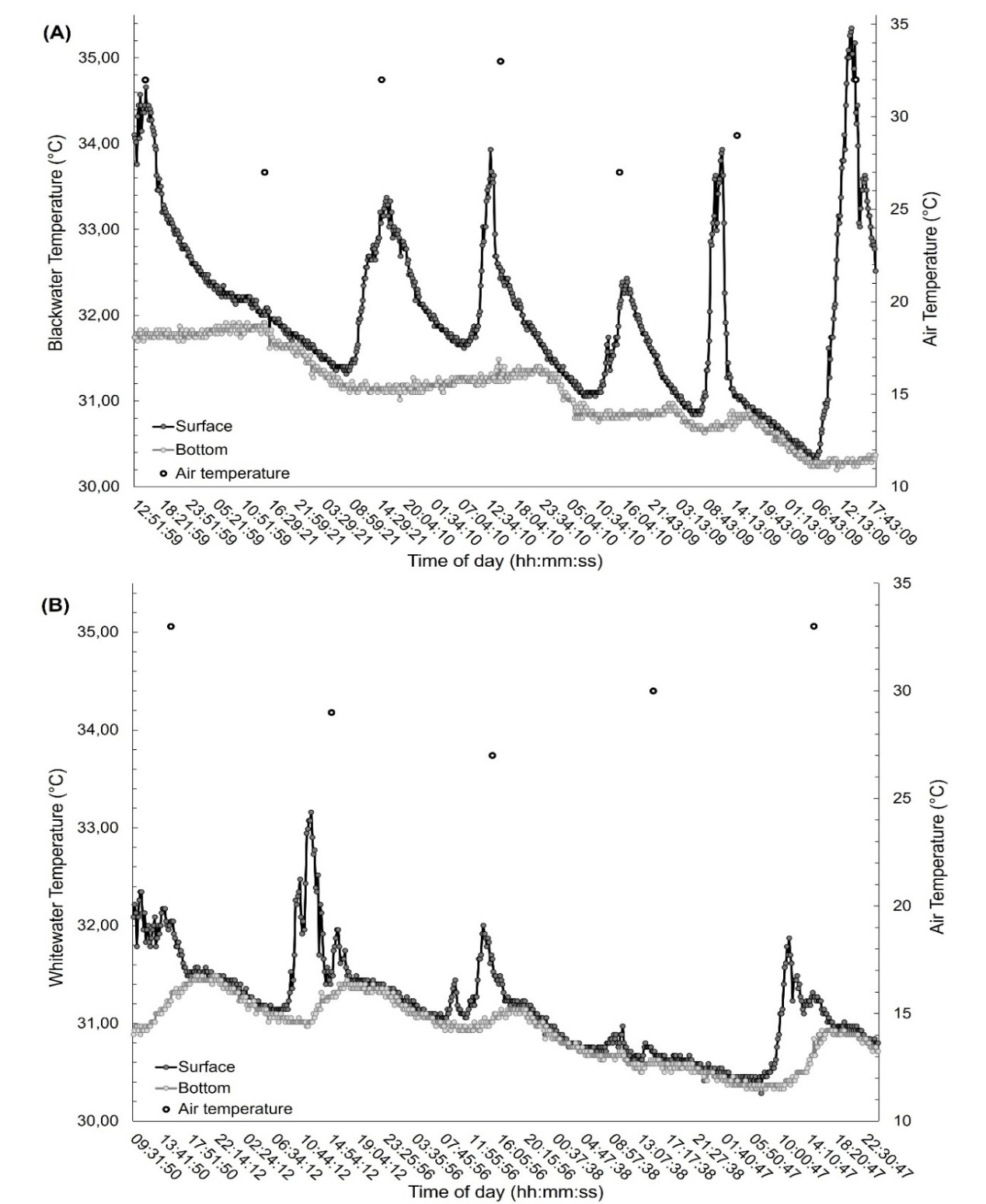


**Figure S1.** Water temperature variation throughout the day, measured at the surface (30 cm deep) and at the bottom (5.8 meters deep) of a (A) blackwater river and (B) a whitewater river. Data were collected in the blackwater river (Anavilhanas Archipelago) between November 22-28, 2023, and in the whitewater river (Amazon River close to Manaquiri city) between November 30 and December 5, 2023. The secondary axis shows the daily average air temperature during each period. Data first published by Braz-Mota S, Duarte RM, Val AL (2025).

**S2**


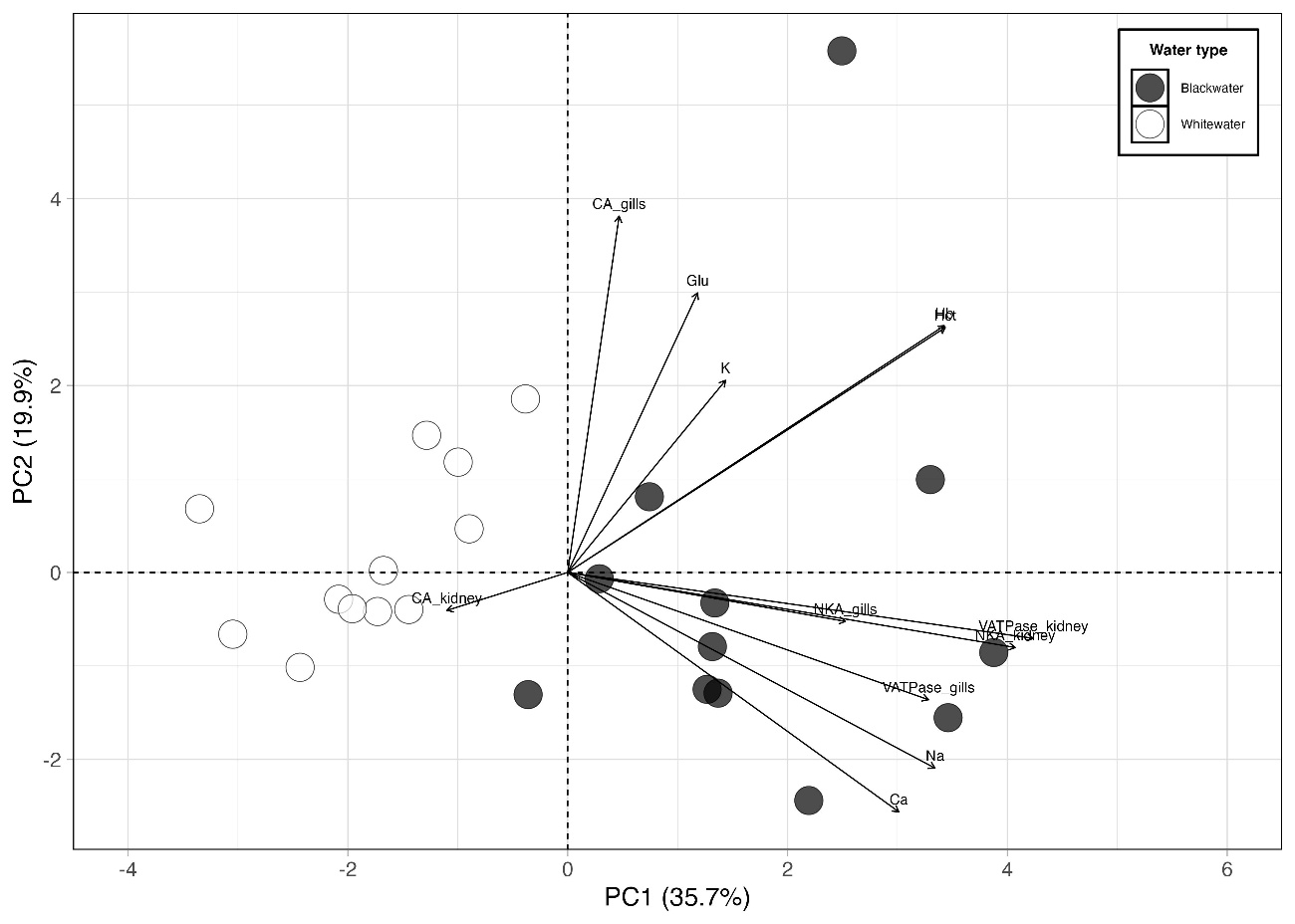


**Figure S2.** Principal component analysis (PCA) of physiological and biochemical responses of *Triportheus albus* (N=12) collected in Rio Negro blackwater (black dots) and in Rio Solimões whitewater (white dots). The figure shows the explained variation in data in the first two axis (PC) extracted by PCA and the loading values of each variable in PCs. Note that divided the data set into 2 well defined clusters by type of water in PC1 indicating that parameters such Hct, [Hb], Na, Ca and NKA and NEM-sensitive ATPase (VATPase) in both gills and kidneys were significantly higher in fish from Rio Negro blackwater, than seen in those collected from Rio Solimões whitewater.
